# Contemporary Trends and Predictors Associated with Adverse Pathological Upstaging Among Non-Metastatic Localized Clinical T2 Muscle-Invasive Bladder Cancers Undergoing Radical Cystectomy: Outcomes from a Single Tertiary Centre in the United Kingdom

**DOI:** 10.3390/cancers17091477

**Published:** 2025-04-27

**Authors:** Francesco Del Giudice, Yasmin Abu-Ghanem, Rajesh Nair, Elsie Mensah, Jonathan Kam, Youssef Ibrahim, Mohamed Gad, Kathryn Chatterton, Suzanne Amery, Romerr Alao, Ben Challacombe, Mohammed Hegazy, Felice Crocetto, Valerio Santarelli, Jan Łaszkiewicz, Bernardo Rocco, Alessandro Sciarra, Benjamin I. Chung, Ramesh Thurairaja, Muhammad Shamim Khan

**Affiliations:** 1 Department of Maternal-Infant and Urological Sciences, “Sapienza” University of Rome, Umberto I Hospital, 00185 Rome, Italy; 2 Department of Urology, Stanford University School of Medicine, Stanford, CA 94304, USA; 3 Guy’s and St. Thomas’ NHS Foundation Trust, Guy’s Hospital, London SE1 7EH, UKsuzanne.amery@gstt.nhs.uk (S.A.); romerr.alao@gstt.nhs.uk (R.A.);; 4 Department of Neurosciences, Reproductive Sciences and Odontostomatology, University of Naples “Federico II”, 80131 Naples, Italy; 5 University Center of Excellence in Urology, Wroclaw Medical University, 50367 Wroclaw, Poland; 6 Deptartment of Urology, Fondazione Policlinico Universitario A. Gemelli IRCCS, Università Cattolica del Sacro Cuore, 00136 Roma, Italy

**Keywords:** muscle-invasive bladder cancer (MIBC), radical cystectomy (RC), pathological upstaging, clinical predictors, robot-assisted radical cystectomy (RARC)

## Abstract

**Introduction**: Radical cystectomy (RC) is the gold standard for urothelial cT2-4a, N0, M0 muscle-invasive bladder cancer (MIBC). However, bladder-sparing strategies (BSS) such as Trimodality Therapy (TMT) have emerged as alternative treatments for a select group of localized muscle-confined (cT2) urothelial bladder cancers. Accordingly, reliable preoperative staging and a reliable risk factor assessment linked to pathological upstaging play a key role in adequate counselling and patient selection for BSS. **Patients and Methods**: cT2 MIBC patients undergoing RC at our institution from 2014 to 2024 were reviewed. Preoperative staging modalities, demographics, and tumour and patient characteristics were assessed. Multivariable logistic regression was applied to explore the relative effect of confounders on any pathological upstaging from robot-assisted or open RC specimens. Subgroup analysis according to the local upstaging (>pT2) or nodal dissemination (pN+) was also performed. **Results**: *N* = 275 RCs were included (73.5% males, 26.5% females). Upstaging was documented in *n* = 141 (51%) cases. Of these, *n* = 125 (45.5%) were upstaged locally (>pT2) and *n* = 35 (23%) yielded pN+ disease. Preoperative parameters like gender, the number of TURBTs, previous BCG exposure, and concomitant CIS did not significantly influence the risk of any kind of upstaging (*p* > 0.05). At multivariable analysis, neoadjuvant chemotherapy (NAC) and multi-disciplinary team (MDT) discussion were found protective (odds ratio [OR]: 0.4, 95%CI 0.2–0.7, *p* = 0.001 and OR: 0.51, 95%CI 0.2–0.9, *p* = 0.01). Preoperative FDG-PET assessment yielded higher risk for later pN upstaging (OR: 1.8, 95%CI 1–3, *p* = 0.05). HG/G3 features at TURBT along with mixed/pure histology variants in RC specimens were the most relevant independent predictors for both any and pT upstaging (OR: 4.3, 95%CI 1–34, *p* = 0.04 and OR: 2.3, 95%CI 1.1–4.6, *p* = 0.02 for any upstaging and OR: 5.6, 95%CI 1.3–36, *p* = 0.02 and OR: 2.5, 95%CI 1.3–5, *p* = 0.01 for pT upstaging, respectively). **Conclusions**: In this study, over half of the patients undergoing RC for cT2 were upstaged at the final pathology. Therefore, adequate counselling and examining the non-conventional criteria for prognosis is mandatory in the contemporary era of bladder-preservation strategies.

## Simple Summary

In this article, we reviewed 275 cT2N0M0 bladder cancer patients who underwent radical cystectomy at our institution from 2014 to 2024. The aim was to assess the preoperative and intraoperative predictors of pathological upstaging (>pT2 or >pN+). We found that high grade carcinomas at TURBT and variant histology were independent risk factors for upstaging, while multidisciplinary management and neoadjuvant chemotherapy were found to be protective. In addition, clinical lymph node staging with FDG-PET imaging was found to yield a higher risk of pN+ at final pathological analysis. An accurate preoperative staging and a precise understanding of risk factors for pathological upstaging are fundamental in the era of bladder-sparing strategies.

## 1. Introduction

Bladder cancer (BC) is the ninth most common malignancy worldwide and the sixth most common in Europe, with an age-standardized incidence of 17.7 per 100,000 [1]. It is almost three times more common in the males, with a mortality rate of 5.2 per 100,000 among European men [2,3]. Radical cystectomy (RC) with or without neo-adjuvant chemotherapy (NAC) is the standard of care, as a curative intervention for non-metastatic, muscle-confined, or locally advanced BC. Also, it is indicated among high- or very high-risk non-muscle-invasive BC (NMIBC) according to the European Association of Urology (EAU) guidelines [4].

cT2N0M0-MIBC patients are a particularly challenging population. Despite the tumour’s infiltration of bladder muscle, it is still in a relatively early stage of progression in these patients. At this stage, multiple therapeutic options can be offered with curative intent and good survival outcomes. Particularly, after adequate consultation and multidisciplinary team (MDT) assessment, patients might opt for bladder-sparing strategies (BSSs) such as Trimodality Therapy (TMT) to avoid the negative impact of RC on quality of life (QoL) [5,6]. The current guidelines contraindicate TMT in patients with multifocal or bulky tumours, extensive carcinoma in situ (CIS), locally advanced disease (i.e., cT3), or in the case of lymph node involvement [7]. Even when RC is selected as the primary therapeutic option, MDT-guided choices, such as undergoing NAC, the extent of the lymphadenectomy, the best urinary diversion (UD), or performing a prophylactic urethrectomy (PU), are strictly dependent on tumour stage [7]. In this scenario, adequate patient selection and precise clinical staging is mandatory to provide the best therapeutic options without compromising oncological safety. According to the latest guidelines, the clinical staging of bladder cancer currently comprises a combination of physical examination, including bimanual examination, axial abdominal/chest imaging, and TURBT [7]. However, the accuracy of clinical staging in BC is particularly low, and multiple studies have reported a discrepancy in up to 50% of cases between clinical and pathologic staging [8,9,10]. Variant histology, female gender, high-grade tumour, and lymphovascular invasion (LVI) have been described as risk factors for pathological upstaging [11,12], but their relative contribution to the risk of pathologic specimen upstaging is not well defined.

After RC, upstaging at the final pathology is potentially a reflection of poor preoperative clinical staging. Moreover, in those who received neoadjuvant chemotherapy (NAC), the finding of residual cancer suggests a poor response to the regimen of choice [13]. In this study, we aimed to identify independent perioperative clinical and pathological predictors of adverse pathological upstaging in patients with clinically localized T2 MIBC undergoing RC. Modelling the impact of these factors may provide valuable guidance for therapeutic decision-making, particularly for patients considering bladder-sparing approaches.

## 2. Patients and Methods

### 2.1. Study Cohort and Radical Cystectomy Registry

Patients with histologically confirmed non-metastatic (i.e., cN0, M0) localized clinical T2 MIBC who underwent RC at our institution from 2014 to 2024 were reviewed. Patients’ demographic, anthropometric, and clinical characteristics (i.e., age, gender, body mass index [BMI], American Society of Anaesthesiologists [ASA] score, and Charlson Comorbidity Index [CCI, with ≥5 defined as “significantly comorbid”) were annotated. All patients were offered RC with or without platinum-based neoadjuvant chemotherapy (NAC) according to the EAU guideline recommendations. Each patient enrolled in the study signed an informed consent form before undergoing RC according to the European Association of Urology (EAU) and Good Clinical Practice (GCP) guidelines, and the ethical principles of the latest version of the Declaration of Helsinki.

The inclusion criteria were the absence of lymph node involvement and distant metastasis at clinical staging, with histological urothelial or mixed-histology diagnosis of both primary or recurrent/progressing BC obtained via at least one prior staging TURBT before RC. All RC interventions were considered the primary surgical therapeutic option with curative intent. The exclusion criteria were previous External Beam Pelvic Radiation Therapy (EBRT) for pelvic solid malignancies and RC performed for other indications rather than BC, including palliative or functional intent (e.g., radiation cystitis, palliative urinary diversion, chronic pelvic pain syndrome, etc.). Clinically positive lymph nodes (i.e., pN 1-2), locally advanced disease (i.e., cT3-T4a), or primary high-risk NMIBCs (i.e., pTis-pT1) were not included. Each patient had a pre-operative staging Computerized Tomography (CT) and a pre-TURBT pelvic multiparametric magnetic resonance imaging (mpMRI) and/or a fluorodeoxyglucose positron emission tomography (FDG-PET) in addition to the CT, in selected cases according to the final consensus from the Urothelial Multidisciplinary Meeting (MDT) recommendations. The clinical stage was assigned via a single or combination of diagnostic tool assessments such as preoperative cystoscopy, TUR-based histology, or systemic/local imaging assessment (i.e., mpMRI, CT, FDG-PET).

### 2.2. Surgical Procedure and Pathological Upstaging Definition

Patients underwent open or robot-assisted RC (ORC, RARC) procedures led by a team of three experienced consultants (MSK, RT, RN) in the setting of a UK-certified Senior Robotic Clinical Fellowship programme on urothelial malignancies. Either intracorporeal or extracorporeal Ileal Conduit (IC), Ureterocutaneostomy (UCS), or Orthotopic Neobladder were performed as appropriate following adequate MDT assessment and patient counselling. All procedures were carried out at the same institution using the same standardized technique for RC as previously reported [14]. Pelvic lymphadenectomy (PLND) was performed with either a standard or an extended template in selected cases. Operative time, intra- or peri-operative complications, readmissions, and blood transfusion rates as a surrogate of a major bleeding event were recorded.

The final histology and pathological stage were reported according to the American Joint Committee on Cancer (AJCC) guidelines, with the RC specimens analyzed by our institutional experienced uropathologists with >20 years of experience in BC. The pathology report included the pT and pN stage, tumour grade, histological variants, and surgical margin, as well as concomitant CIS and lymph vascular invasion (LVI). cT2-MIBC patients were considered upstaged when the final pathological report demonstrated a pT stage equal to or higher than pT3, or when the pathological lymph node specimen yielded positivity (i.e., pN 1-2).

### 2.3. Statistical Analyses

Statistical analyses, along with the reporting and interpretation of the results, were conducted according to the previously described methodology and consisted of four separate analytical steps [15].

Firstly, descriptive statistics were used to summarize the pertinent study information stratified according to the pathological upstaging status of RC specimens. The number of cases, percentages, and median and interquartile (IQR) ranges were adopted to depict the numerosity of the samples. The association between variables was tested using the Pearson Chi-square test or Fisher’s exact test when appropriate. A Mann–Whitney test or ANOVA one-way test was adopted when analyzing quantitative data and pairwise intergroup comparisons of variables.

Secondly, a set of univariable regression models was developed to explore the effect estimate of each clinical, demographic, and/or pathological predictor on final pathological upstaging. The analysis was focused on but not limited to common BC- and patient-related variables including age, gender, number of TUR procedures, previous BCG exposure, tumour grade, and concomitant CIS status. A second group of MDT-influenced covariates was also explored, including the execution of level II preoperative staging modalities such as mpMRI, FDG-PET, or NAC administration. Perioperative confounders consisted of surgical approach, intra-operative complications, lymph node templates, pathological tumour stage, and variant histology. We then applied multivariable logistic regression modelling using stepwise regression (forward selection) by selecting those predictive variables that were significant upon univariate analysis to identify independent predictors for histological upstaging in the RC specimen report. Finally, the pT and degree of pN status, including lymph node retrieval density and relative percentage of histological variants, were plotted, using the locally weighted scatter plot smoother (LOWESS) function against the multivariable-adjusted predicted probability models for any pathological upstaging. This was meant to graphically depict the influence of the spectrum of quantitative clinical predictor variation on the pre-established endpoint.

The statistical analysis was performed using Stata version 18.1 (Stata Corporation, College Station, TX, USA), with statistical significance set as *p* < 0.05. The enter and remove significance limits were set at *p* = 0.05 and *p* = 0.10, for univariable or multivariable models, respectively.

## 3. Results

### 3.1. Study Cohort Characteristics According to Final Path Upstaging

The baseline characteristics of the entire cohort are shown in Table 1. A total of 275 patients were included in the study, 202 (73.5%) were males and 73 (26.5%) females. The median age was 69 years (IQR 55–78). The median BMI, CCI, and ASA scores were 27.4 (IQR 24–31), 5 (2–8), and 2 (1–3), respectively. Most patients (*n* = 235, 85.5%) underwent a single TURBT prior to cystectomy, while 14.5% (*n* = 40) of cases underwent two or more previous TURBTs. Concomitant CIS in any of the previous TURBTs was found in 28.4% (*n* = 78) of cases. Most patients underwent RC with a diagnosis of high-grade (HG) BC at one of the previous TURBTs (*n* = 260, 95.6%), and only 12 of the included patients (4.4%) had an LG tumour at TURBT. A total of 35 (12.7%) patients were previously exposed to intravesical BCG. In all cases, a CT scan was performed prior to surgery (<3 months prior to the operation). Additionally, a total of 35 (12.7%) patients also underwent mpMRI, and 76 (27.6%) an FDG-PET scan after initial CT staging where there was uncertainty about local stage (mpMRI) or metastatic disease (FDG-PET) after MDT discussion. Prior to undergoing RC, 223 (81.1%) cases were discussed in the MDT session and NAC was administered in 88 (32%) patients.

A total of 79 (28.4%) patients underwent RC with an open approach, while RARC was the approach of choice for 196 (71.6%) patients (of note, the relatively high number of patients who underwent an RC with an open approach is to be largely attributed to our center’s participation in the IROC trial and the consequential randomization of patients to an open or robotic approach during the period of activity of the study [16]). PLND was performed as the per standard template in 211 (76.7%) patients, and with an extended template in 64 (23.3%). Regarding reconstruction, an Orthotopic Neobladder was the diversion of choice in 5.8% (*n* = 16) of patients. Ileal Conduit (IC) was the most frequently performed diversion (253 cases, 92% of patients, 200 intracorporeal and 53 extracorporeal), and Ureterocutaneostomy (UCS) in 6 cases, 2.2%). The median Estimated Blood Loss (EBL) was 400 mL (300–625 mL) and intraoperative complications occurred in 23 cases (8.3%), mostly of the Clavien–Dindo grades I (21.5%) and II (53.2%). The median postoperative discharge day was day 7 (IQR 4–11).

**Figure 1:**
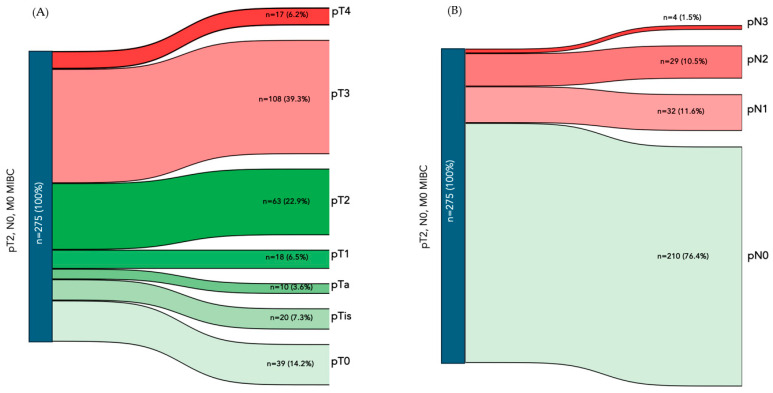
Sankey diagram depicting final pT (**A**) and pN (**B**) stage rates of the initially homogeneous cT2N0M0 population.

At the final pathology, 63 patients (22.9%) had stage-pT2 disease. A total of 125 (45.5%) patients had pathological stage pT3 or pT4 (*n* = 108 pT3 and *n* = 17 pT4) and 87 (31.6%) were pT1, pTis, or lower. A complete pathological response to NAC (ypT0) was achieved in 39 (14.2%) patients from the total cohort. Positive lymph nodes (pN+) were found in 65 (23.6%) patients. Most tumours were HG tumours, with only one (0.4%) LG carcinoma at final pathological analysis. A total of 49 (17.8%) patients had a histological variant, either mixed or pure. Overall, 141 patients (51.3%) had either pT upstaging (>stage pT2) or positive lymph nodes (pN upstaging), or both, at final pathological analysis (Figure 1).

### 3.2. Any Pathological Upstaging at RC Specimen

The risk assessment for the overall, pT, and pN upstaging is shown in Table 2. At univariate analysis, preoperative parameters such as gender, number of TURBT procedures, previous BCG exposure, and concomitant CIS did not significantly influence the risk of upstaging at the final pathology. HG tumours at TURBT had a significantly higher risk of being upstaged (OR = 2.1, 95%CI 1–5.7, *p* = 0.04). Staging tools (MRI or PET-CT), surgical technique (cystectomy technique, whether open or robotic), lymph node excision template (standard vs. extended), and diversion type did not impact the risk of overall upstaging. Patients who underwent NAC had a significantly lower risk (OR = 0.4, 95%CI 0.2–0.7, *p* = 0.001) of upstaging. Similarly, being discussed in an MDT session nearly halved the risk of overall upstaging (OR = 0.51, 95%CI 0.2–0.9, *p* = 0.01). At the final pathology, the presence of a histological variant significantly increased the risk of upstaging (OR = 1.82, 95%CI 1.1–3.4, *p* = 0.04).

### 3.3. Predictors for Only pT Upstaging

When considering only local (pT) upstaging as a variable of interest, preoperative parameters like sex, the number of TURBTs, previous BCG exposure, and concomitant CIS did not influence the risk of pT upstaging at the final pathology. Likewise, imaging for staging (MRI or PET-CT) and surgical technique (cystectomy technique, lymph node excision template, and diversion type) did not impact the risk of local upstaging. Patients who underwent NAC and those who were discussed in an MDT session had a significantly lower risk (OR = 0.41, 95%CI 0.2–0.7, *p* = 0.001 and OR = 0.6, 95%CI 0.3–1, *p* = 0.02) of local upstaging. HG at TURBT and variant histology were confirmed to be risk factors for pT upstaging, with a higher correlation than overall upstaging (OR = 5.6, 95%CI 1.2–37, *p* = 0.002 and OR = 4, 95%CI 2.5–6.5, *p* < 0.001).

### 3.4. Predictors for Only pN Upstaging

Sixty-five patients were found to have at least one positive lymph node (pN+) at final pathological analysis. Preoperative parameters like sex, the number of TURBTs, previous BCG exposure, and concomitant CIS did not significantly influence the risk of pN upstaging at final pathology. No correlation could be assessed between grade at cystectomy and lymph node positivity. Regarding preoperative locoregional staging tools, patients who underwent FDG-PET for systemic and locoregional staging were found to have a higher risk of regional (pN) upstaging at the final pathology, but the difference did not reach the level of significance on univariate analysis (OR 1.6; 95%CI 0.9–3, *p* = 0.1). Patients who underwent NAC and MDT discussion had a significantly lower risk (OR = 0.3, 95%CI 0.1–0.6, *p* = 0.001 and OR = 0.4, 95%CI 0.2–0.8, *p* = 0.005) of regional upstaging, and no significant correlation was found between the presence of a histological variant and lymph node invasion (OR = 1.5, 95%CI 0.8–3, *p* = 0.2), mostly due to the low sample number.

### 3.5. Multivariable Regression Modelling for Independent Upstaging Predictors

**Figure 2:**
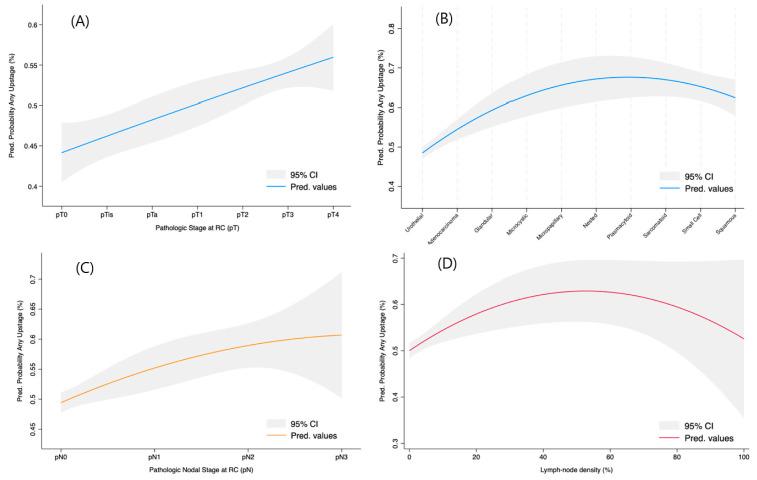
Locally weighted scatter plot smoother (LOWESS) function depicting the influence of pT status (**A**), pN status (**B**), different variant histologies (**C**), and positive lymph node density (**D**) on the multivariable-adjusted predicted probability models for any pathological upstaging.

Results of multivariate analysis are shown in Table 3. At multivariate analysis, gender, concomitant CIS, previous BCG exposure, and pelvic MRI for local staging were not found to be independent risk factors for any upstaging. NAC and pelvic MDT discussion were found to be protective for all kinds of upstaging (respectively, OR = 0.4, 95%CI 0.2–0.7 and OR = 0.5, 95%CI 0.3.0–9 for overall upstaging, OR = 0.4, 95%CI 0.2–0.7 and OR = 0.5, 95%CI 0.3–0.9 for pT upstaging, and OR = 0.3, 95%CI 0.1–0.6 and OR = 0.4, 95%CI 0.2–0.9 for pN upstaging). In multivariate regression, undergoing FDG-PET for locoregional staging significantly increased the risk of pN upstaging at the final pathology (OR = 1.8, 95%CI 1–3, *p* = 0.05). A high grade of the tumour at TURBT and a variant histology at RC were found to be independent risk factors for overall and pT upstaging (OR = 4.3, 95%CI 1–17 and OR = 2.3, 95%CI 1.1–4.6 for total upstaging and OR = 5.6, 95%CI 1.3–36 and OR = 2.5, 95%CI 1.2–5.1 for pT upstaging). Figure 2 provides a graphical representation of the upstaging probability on the basis of different pathological characteristics.

## 4. Discussion

Previous evidence has highlighted the mismatch between preoperative clinical staging via different types of assessment modalities and the postoperative final RC pathology [8,9]. An inadequate preoperative clinical stage may indeed delay definitive interventions and lead to errors in treatment recommendations, ultimately negatively impacting survival outcomes [17]. Recently, with the growing adoption of NAC and TMT, precise case selection in terms of both tumour and patient characteristics has become critically relevant to effectively achieve optimal survival outcomes [3,7].

In our study, we interrogated our large single-institution cohort to determine the preoperative and intraoperative clinical/pathological features associated with upstaging at the final RC pathology. Of note, concomitant CIS in the TUR specimen did not significantly impact the risk of upstaging. However, according to historical EORTC and NMIBC EAU guidelines, this is well known as an adverse prognostic factor for later disease progression and reduced survival chances [18]. On the other hand, by examining only cT2-MIBC cohorts, previous evidence demonstrated conflicting results regarding the prognostic significance of CIS. In a prospective multicentre study of 196 cT2-MIBC patients, no correlation was identified between concomitant CIS and upstaging [19], while the results of a series of 1968 RC patients found concomitant CIS to be an independent predictor for upstaging [20]. Given this discrepancy, the association between the presence and extent of CIS and risk of upstaging needs to be better defined in future studies to offer the best therapeutic option for this particular class of patients. In our study, pelvic MRI was found to be a reliable preoperative locoregional staging tool. On the other hand, patients who underwent PET-CT were found to have a higher risk of non-detected lymph node involvement (i.e., false negatives). These results might appear surprising and in contrast to previous research, reporting a higher risk of clinical upstaging than downstaging in patients undergoing RC [21,22]. However, since only cN0M0 patients were reviewed in the present study, the risk of clinical upstaging with the different imaging tools adopted and the total diagnostic accuracy could not be determined and was out of the scope of the study. While it is accepted that FDG-PET/CT is superior for the detection of lymph node invasion compared to CT, no single staging method provided optimal diagnostic accuracy [21]. For this reason, until a dedicated imaging technique with a specific tracer is available, such as Prostate-Specific Membrane Antigen (PSMA)-PET for prostate cancer, multimodality assessment staging is preferred to increase accuracy in those patients who are eligible for BSS [23].

NAC prior to cystectomy has been recommended for eligible cT2-T4N0M0 patients since 2008. Considering this recommendation, the rate of patients who underwent NAC in the present study (32%) might appear to be relatively low. However, these results do not substantially differ from those reported in other population-based studies [24,25]. Moreover, the role of NAC as a predictor of lower Cancer-Specific Mortality (CSM) in the organ-confined stage (namely cT2N0M0) is less corroborated than non-organ-confined disease, and just recently received formal validation [25,26]. Consequently, while the vast majority of cases were discussed in an MDT session, it is not surprising that a high percentage of patients, after being offered NAC according to the latest guidelines, refused or were not fit to undergo NAC in light of uncertain survival. Of note, it is important to reiterate that those patients who underwent NAC were found to have a significantly lower risk of local or regional upstaging, once more corroborating the importance of adequate perioperative systemic therapies. On the other hand, *n* = 32 of the patients (36.4%) undergoing NAC were upstaged, re-confirming the well-known limitations of NAC’s non-response rates, which in our case, are closer to the higher end of those reported in the literature (8–41%) [27,28]. Suboptimal NAC and longer intervals before radical cystectomy are the main risks associated with a poor response to NAC [29]. While it is true that NAC and chemotherapy regimens adopted during TMT for MIBC differ, a more thorough understanding of the clinical and pathological risk factors associated with upstaging after NAC could help better define predictors of poor outcomes of BSS. As previously reported, different factors have been associated with pathological upstaging for RC, and many of those have been evaluated in the setting of platinum-based NAC [8,9,10]. Immunotherapy-based systemic regimens are currently the standard of care for metastatic BC [30]. Moreover, EBRT has been demonstrated to enhance the cross-presentation of tumour antigens and the upregulation of PD-L1 expression, further validating the adoption of Immune Checkpoint Inhibitors (ICI) in TMT [31,32]. Currently, only platinum-based regimens are approved by the international guidelines for NAC prior to RC. Accordingly, all patients included in the present study underwent platinum-based regimens. Growing research is focusing on expanding the indication of immunotherapy to this setting [33]. In this scenario, the factors currently associated with poor responses to ICI might become risk factors of poor responses to novel NAC regimens, and ultimately of RC upstaging [34].

Another critical step of our article is highlighting how adequate preoperative MDT discussion reduced the risk of upstaging at the final pathology. These results could partially be explained by the higher rate of MDT patients who underwent NAC, but even after adjustment with multivariate analysis, the difference remained significant. The indication for in-depth additional imaging, or a second inspection of the already-available radiological examinations in the setting of an MDT session, attended by experienced uroradiologists, has surely contributed to a more precise stage definition. In any case, as treatment options grow, the number of patients being submitted for an MDT discussion is increasing, especially in larger and tertiary centres [35]. Regarding pathological predictors, we found that both a HG tumour at TURBT and the presence of a variant histology in RC specimens were significantly and independently associated with upstaging, particularly with pT upstaging. Only *n* = 1 LG patient was confirmed at final pathological analysis of the RC specimen. This discrepancy could be attributed to an upgrading of BC during the time elapsed between TURBT and RC or, most likely, to an uncomplete or suboptimal primary resection. Results from the TURBT specimen might not have been representative of the whole tumour, especially in the case of mixed grades or histology. Previous studies reported lower response rates to NAC (both in terms of pathological downstaging and overall survival) for BCs with a variant histology compared to conventional urothelial carcinoma (UC) [36,37]. This is consistent with the poor responses to chemotherapy demonstrated by some variant histologies, like plasmacytoid, sarcomatoid, and pure squamous cell carcinoma [38]. However, the results remained significant even after adjustment for NAC at multivariate analysis, suggesting the role of variant histology as an independent risk factor for pathological upstaging. The poor responses to chemotherapy and the time-sensitive risk of upstaging and progression suggest that caution is necessary when considering patients diagnosed with a different histological type of BC for BSS. Nevertheless, few of the available studies have evaluated the impact of variant histology on TMT outcomes, and the results are still not able to determine whether specific histological variants should be included or omitted from TMT, or should perhaps be offered an alternative personalized BSS protocol [39,40].

Our study is not devoid of limitations. First, it is a retrospective study with a relatively small sample size. Second, more specific data (such as cycles, dosage and specific regimens of NAC, or type and percentage of various histological variants, etc.) were not ready for review at the time of analysis. Lastly, we were not able to evaluate the impact of the included variables on survival outcomes, as the survival analyses were not performed in this study.

## 5. Conclusions

In our study, we evaluated the preoperative clinical and pathological factors and intraoperative variables associated with upstaging at the final pathology in a cohort of 275 cT2-MIBC patients undergoing RC. We found that lymph node staging with FDG-PET-CT increased the risk of upstaging at final histological analysis, and that MDT and NAC were independent protective factors. Our results suggest that patients with variant histologies and HG tumours are at higher risk of upstaging when compared to pure urothelial carcinomas and LG tumours, even when undergoing NAC. If we aim for increasing the number of patients undergoing NAC or bladder-preserving strategies, future research should focus on the precise determination of the clinical and pathological predictors of upstaging for this heterogeneous class of patients.

## Figures and Tables

**Table 1:** Demographic, perioperative, and pathological characteristics of the study cohort reviewed according to pathological non-upstaging vs. upstaging in radical cystectomy specimens (i.e., ≤pT2, pN0 vs. >pT2, >pN0).

	Total	pT/N Non-Upstaging (i.e., ≤pT2, pN0)	pT/N Upstaging (i.e., >pT2, >pN0)	*p* Value
**Sample Size**, *n* (%)	*N* = 275	*N* = 134 (47.2%)	*N* = 141(51.3%)	
**Gender**, *n* (%)				0.7
Male	202 (73.5%)	100 (74.6%)	102 (72.3%)	
Female	73 (26.5%)	34 (25.4%)	39 (27.7%)	
**Age**, median (IQR)	69 (55–78)	68 (55–76)	68 (55–78)	0.5
**BMI**, median (IQR)	27.4 (24–31)	27.5 (24–31)	27.3 (24.1–30)	0.8
**CCI**, median (IQR)	5 (2–8)	5 (2–8)	5 (2–7)	0.8
**ASA**, median (IQR)	2 (1–3)	2 (1–3)	2 (1–3)	0.9
**No. of previous TURBTs**, *n* (%)				0.9
1	235 (85.5%)	115 (85.8%)	120 (65.2%)	
≥2	40 (14.5%)	19 (14.2%)	21 (14.8%)	
**Concomitant CIS**, *n* (%)				0.8
No	197 (71.6%)	97 (72.4%)	100 (71%)	
Yes	78 (28.4%)	37 (27.6%)	41 (29%)	
**TURBT Grade**, *n* (%)				0.02
Low grade	12 (4.4%)	10 (7.7%)	2 (2.2%)	
High grade	260 (95.6%)	123 (92.5%)	137 (97.8%)	
**Previous BCG exposure**, *n* (%)				0.48
No	240 (87.3%)	115 (85.8%)	125 (88.6%)	
Yes	35 (12.7%)	19 (14.2%)	16 (11.4%)	
**mpMRI**, *n* (%)				0.98
No	240 (87.3%)	117 (87.3%)	123 (87.3%)	
Yes	35 (12.7%)	17 (12.7%)	18 (12.7%)	
**FDG-PET**, *n* (%)				0.58
No	199 (72.4%)	99 (68.4%)	100 (71%)	
Yes	76 (27.6%)	35 (31.6%)	41 (29%)	
**MDT discussion**, *n* (%)				0.01
No	52 (18.9%)	17 (12.7%)	35 (24.8%)	
Yes	223 (81.1%)	117 (87.3%)	106 (75.2%)	
**NAC**, *n* (%)				0.001
No	187 (68%)	78 (58.2%)	109 (78.3%)	
Yes	88 (32%)	56 (41.8%)	32 (22.7%)	
**RC surgical approach**, *n* (%)				0.57
ORC	79 (28.4%)	40 (29.9%)	39 (27.7%)	
RARC	196 (71.6%)	94 (70.1%)	102 (72.3%)	
**PLND**, *n* (%)				0.5
Standard	211 (73%)	101 (75.4%)	110 (78%)	
Extended	64 (23%)	33 (24.6%)	31 (22%)	
**Urinary diversion**, *n* (%)				0.5
UCS	6 (2.2%)	3 (2.2%)	3 (2.1%)	
IC intracorporeal	200 (72.7%)	95 (70.9%)	105 (74.5%)	
IC extracorporeal	53 (19.3%)	26 (19.4%)	27 (19.1%)	
Neobladder	16 (5.8%)	10 (7.5%)	6 (4.3%)	
**Any intraop. complications**, *n* (%)				0.65
No	252 (91.7%)	123 (91.8%)	129 (91.5%)	
Yes	23 (8.3%)	11 (8.2%)	12 (8.5%)	
**Clavien–Dindo grade**, *n* (%)				0.4
Grade I	5 (21.5%)	2 (18.2%)	3 (25%)	
Grade II	13 (53.2%)	7 (63.6%)	6 (50%)	
Grade III	4 (17.4%)	2 (18.2%)	2 (16.6%)	
Grade IV	1 (4.3%)	0	1 (8.3%)	
Grade V	0	0	0	
**Length of stay**, median (IQR)	7 (4–11)	7 (4–11)	8 (5–11)	0.5
**pT stage**, *n* (%)				<0.001
pT0	39 (14.2%)	38 (56.1%)	1 (0.7%)	
pTa	10 (3.6%)	10 (14.9%)	0 (0%)	
pTis	20 (7.3%)	17 (25.4%)	3 (2.1%)	
pT1	18 (6.5%)	15 (23.6%)	3 (2.1%)	
pT2	63 (22.9%)	54 (81.1%)	9 (6.4%)	
pT3	108 (39.3%)	0 (0%)	108 (76.6%)	
pT4	17 (6.2%)	0 (0%)	17 (12%)	
**pN stage**, *n* (%)				<0.001
pN0	210 (76.4%)	134 (100%)	76 (53.9%)	
pN1	32 (11.6%)	0 (0%)	32 (22.7%)	
pN2	29 (10.5%)	0 (0%)	29 (20.6%)	
pN3	4 (1.5%)	0 (0%)	4 (2.8%)	
**Grade at RC**, *n* (%)				NA
Low-grade	1 (0.4%)	1 (0.8%)	0	
High-grade	272 (99.6%)	132 (99.2%)	140 (100%)	
**Surgical Margins**, *n* (%)				<0.001
Negative	256 (93.1%)	133 (99.3%)	123 (87.2%)	
Positive	19 (6.9%)	1 (0.7%)	18 (12.8%)	
**Variant Histology at RC**, *n* (%)				0.05
Absent	226 (82.2%)	117 (87.3%)	109 (77.2%)	
Present	49 (17.8%)	17 (12.7%)	32 (22.7%)	

***n***: number; **TURBT**: trans-urethral resection of bladder tumour; **CIS**: carcinoma in situ; **BCG**: Bacillus Calmette-Guérin; **mpMRI**: multiparametric magnetic resonance imaging; **FDG-PET**: fluorodeoxyglucose positron emission tomography; **MDT**: multidisciplinary team; **NAC**: neoadjuvant chemotherapy; **RC**: radical cystectomy; **ORC**: open radical cystectomy; **RARC**: robot-assisted radical cystectomy; **PLND**: pelvic lymph node dissection; **UCS**: Ureterocutaneostomy; **IC**: Ileal Conduit; **pT stage**: pathological tumour stage; and **pN stage**: pathological node stage.

**Table 2:** Risk of any upstaging, pT upstaging, or pN upstaging based on multiple predictors.

	Any Upstaging			pT Upstaging			pN Upstaging		
Parameter	OR	95%CI	*p* Value	OR	95%CI	*p* Value	OR	95%CI	*p* Value
**Gender**									
Male	Ref.			Ref.			Ref.		
Female	0.89	0.5–1.5	0.39	0.9	0.5–1.5	0.36	0.9	0.5–1.7	0.5
**No. of previous TURBTs**									
1	Ref.			Ref.			Ref.		
2+	1.06	0.54–2.07	0.5	0.9	0.4–1.7	0.41	1.1	0.5–2.4	0.5
**Concomitant CIS**									
No	Ref.			Ref.			Ref.		
Yes	1.08	0.64–1.82	0.45	0.97	0.6–1.64	0.51	0.9	0.5–1.6	0.4
Grade at TURBT							NA	NA	NA
Low-grade	Ref.			Ref.					
High-grade	2.1	1.5–7	0.04	5.6	1.2–37	0.002			
**Previous BCG exposure**									
No	Ref.			Ref.			Ref.		
Yes	0.78	0.38–1.58	0.3	0.78	0.38–1.6	0.31	1	0.4–2.2	0.6
**mpMRI**									
No	Ref.			Ref.			Ref.		
Yes	1.01	0.5–2.05	0.56	1.01	0.5–2	0.6	0.8	0.3–1.9	0.4
**FDG-PET**									
No	Ref.			Ref.			Ref.		
Yes	1.16	0.7–1.97	0.34	0.83	0.5–1.4	0.3	1.6	0.9–3	0.1
**MDT discussion**									
No	Ref.			Ref.			Ref.		
Yes	0.51	0.2–0.9	0.01	0.6	0.35–0.96	0.02	0.4	0.2–0.8	0.005
**NAC**									
No	Ref.			Ref.			Ref.		
Yes	0.41	0.24–0.7	0.001	0.41	0.24–0.7	0.001	0.3	0.1–0.6	0.001
**RC surgical approach**									
ORC	Ref.			Ref.			Ref.		
RARC	1.17	0.68–1.99	0.33	1	0.6–1.8	0.5	0.9	0.5–1.6	0.4
**Any intraop. complications**									
No	Ref.			Ref.			Ref.		
Yes	0.71	0.16–3.22	0.5	0.9	0.2–4.1	0.6	0.8	0.7–0.8	0.15
**Variant Histology at RC**									
Absent	Ref.			Ref.			Ref.		
Present	1.82	1.1–3.43	0.04	4	2.5–6.5	<0.001	1.5	0.8–3	0.2

**OR**: odds ratio; **95%CI**: 95% confidence interval; **TURBT**: trans-urethral resection of bladder tumour; **CIS**: carcinoma in situ; **BCG**: Bacillus Calmette-Guérin; **mpMRI**: multiparametric magnetic resonance imaging; **FDG**-**PET**: fluorodeoxyglucose positron emission tomography; **MDT**: multidisciplinary team; **RC**: radical cystectomy; **NAC**: neoadjuvant chemotherapy; and **NA**: not available.

**Table 3:** Multivariate regression models showing the risk of any upstaging, pT upstaging, or pN upstaging on the basis of multiple predictors.

	Any Upstaging			pT Upstaging			pN Upstaging		
Parameter	OR	95%CI	*p* Value	OR	95%CI	*p* Value	OR	95%CI	*p* Value
**Gender**									
Male	Ref.			Ref.			Ref.		
Female	0.9	0.5–1.6	0.75	0.9	0.5–1.6	0.7	0.9	0.5–1.8	0.8
**Concomitant CIS**									
No	Ref.			Ref.			Ref.		
Yes	1.2	0.7–2.2	0.5	1.1	0.6–1.9	0.9	1	0.5–2	0.9
**Grade at TURBT**	Ref.			Ref.			NA	NA	NA
Low-grade									
High-grade	4.3	1.1–17	0.04	5.6	1.3–36	0.02			
**Previous BCG exposure**									
No	Ref.			Ref.			Ref.		
Yes	0.7	0.4–1.6	0.4	0.7	0.3–1	0.4	1	0.4–2.4	1
**mpMRI**									
No	Ref.			Ref.			Ref.		
Yes	1	0.43–2.1	0.9	0.9	0.4–2	0.8	0.9	0.3–2.5	0.9
**FDG-PET**									
No	Ref.			Ref.			Ref.		
Yes	1.3	0.7–2.3	0.4	0.9	0.5–1.5	0.6	1.8	1–3.3	0.05
**MDT discussion**									
No	Ref.			Ref.			Ref.		
Yes	0.5	0.3–0.9	0.02	0.5	0.3–0.9	0.02	0.44	0.2–0.9	0.02
**NAC**									
No	Ref.			Ref.			Ref.		
Yes	0.4	0.2–0.7	0.002	0.4	0.2–0.7	0.02	0.3	0.1–0.6	0.001
**Variant Histology at RC**									
Absent	Ref.			Ref.			Ref.		
Present	2.3	1.1–4.6	0.02	2.5	1.3–5	0.01	1.7	0.8–3.6	0.2

**OR**: odds ratio; **95%CI**: 95% confidence interval; **TURBT**: trans-urethral resection of bladder tumour; **CIS**: carcinoma in situ; **BCG**: Bacillus Calmette-Guérin; **mpMRI**: multiparametric magnetic resonance imaging; **FDG**-**PET**: fluorodeoxyglucose positron emission tomography; **MDT**: multidisciplinary team; **RC**: radical cystectomy; and **NAC**: neoadjuvant chemotherapy.

## Data Availability

The data presented in this study are available upon request from the corresponding authors.

## References

[1] Antoni S., Ferlay J., Soerjomataram I., Znaor A., Jemal A., Bray F. (2017). Bladder Cancer Incidence and Mortality: A Global Overview and Recent Trends. Eur. Urol..

[2] Leal J., Luengo-Fernandez R., Sullivan R., Witjes J.A. (2016). Economic Burden of Bladder Cancer Across the European Union. Eur. Urol..

[3] Witjes J.A., Bruins H.M., Carrión A., Cathomas R., Compérat E., Efstathiou J.A., Fietkau R., Gakis G., Lorch A., Martini A. (2023). European Association of Urology Guidelines on Muscle-invasive and Metastatic Bladder Cancer: Summary of the 2023 Guidelines. Eur. Urol..

[4] Pfail J., Lichtbroun B., Golombos D.M., Jang T.L., Packiam V.T., Ghodoussipour S. (2024). The role of radical cystectomy and lymphadenectomy in the management of bladder cancer with clinically positive lymph node involvement. Curr. Opin. Urol..

[5] Smith J.A. (2003). The quality of life in men after radical cystectomy with a continent cutaneous diversion or orthotopic bladder sub-stitution: Is there a difference?. J. Urol..

[6] de Angelis M., Basile G., Scornajenghi C.M., Asero V., Del Giudice F., Moschini M. (2023). Bladder-sparing strategies in patients with clinically localized muscle-invasive bladder cancer. Curr. Opin. Urol..

[7] Kamat A.M., Black P.C. (2021). Bladder Cancer: A Practical Guide.

[8] Bayraktar Z., Gurbuz G., Taşci A.I., Sevin G. (2001). Staging error in the bladder tumor: The correlation between stage of TUR and cys-tectomy. Int. Urol. Nephrol..

[9] Mclaughlin S., Shephard J., Wallen E., Maygarden S., Carson C.C., Pruthi R.S. (2007). Comparison of the clinical and pathologic staging in patients undergoing radical cystectomy for bladder cancer. Int. Braz. J. Urol..

[10] Svatek R.S., Shariat S.F., Novara G., Skinner E.C., Fradet Y., Bastian P.J., Kamat A.M., Kassouf W., Karakiewicz P.I., Fritsche H. (2011). Discrepancy between clinical and pathological stage: External validation of the impact on prognosis in an international radical cystectomy cohort. BJU Int..

[11] Turker P., Bostrom P.J., Wroclawski M.L., van Rhijn B., Kortekangas H., Kuk C., Mirtti T., Fleshner N.E., Jewett M.A., Finelli A. (2012). Upstaging of urothelial cancer at the time of radical cystectomy: Factors associated with upstaging and its effect on outcome. BJU Int..

[12] McFadden J., Tachibana I., Adra N., Collins K., Cary C., Koch M., Kaimakliotis H., Masterson T., Rice K. (2024). Impact of variant histology on upstaging and survival in patients with nonmuscle invasive bladder cancer undergoing radical cystectomy. Urol. Oncol..

[13] van Hoogstraten L.M.C., Man C.C.O., Witjes J.A., Meijer R.P., Mulder S.F., Smilde T.J., Ripping T.M., Kiemeney L.A., Aben K.K.H., BlaZIB Study Group (2023). Low adherence to recommended use of neoadjuvant chemotherapy for muscle-invasive bladder cancer. World J. Urol..

[14] Albisinni S., Orecchia L., Mjaess G., Aoun F., Del Giudice F., Antonelli L., Moschini M., Soria F., Mertens L.S., Gallioli A. (2024). Enhanced Recovery After Surgery for patients undergoing radical cystectomy: Surgeons’ perspectives and recommendations ten years after its implementation. Eur. J. Surg. Oncol. (EJSO).

[15] Santarelli V., Carino D., Corvino R., Salciccia S., De Berardinis E., Krajewski W., Nowak Ł., Łaszkiewicz J., Szydełko T., Nair R. (2024). Surgical Technique and Perioperative Outcomes of the “Sapienza” Urology Residency Program’s Trocar Placement Configuration During Robotic-Assisted Radical Prostatectomy (RARP): A Retrospective, Single-Centre Observational Study Comparing Experienced Attendings vs. Post-Graduate Year I–III Residents as Bedside Assistants. Cancers.

[16] Khetrapal P., Catto J., Ambler G., Ricciardi F., Khan S., Feber A., Dixon S., Williams N., Ahmed I., Charlesworth P. (2022). PD42-02 Results of the Intracorporeal Robotic vs Open Cystectomy (IROC) Multi-Centre Randomised Trial. J. Urol..

[17] Ge P., Wang L., Lu M., Mao L., Li W., Wen R., Lin J., Wang J., Chen J. (2018). Oncological Outcome of Primary and Secondary Muscle-Invasive Bladder Cancer: A Systematic Review and Meta-analysis. Sci. Rep..

[18] Babjuk M., Burger M., Compérat E.M., Gontero P., Mostafid A.H., Palou J., van Rhijn B.W.G., Roupret M., Shariat S.F., Sylvester R. (2019). European Association of Urology Guidelines on Non-muscle-invasive Bladder Cancer (TaT1 and Carcinoma In Situ)—2019 Update. Eur. Urol..

[19] El-Adawy M.S., Ibrahim H., Zanaty F., Kotb S. (2022). Factors related to upstaging of clinical stage T2 organ-confined bladder cancer following radical cystectomy: A multicenter study. Urol. Ann..

[20] Yafi F.A., Aprikian A.G., Chin J.L., Fradet Y., Izawa J., Estey E., Fairey A., Rendon R., Cagiannos I., Lacombe L. (2013). Impact of concomitant carcinoma in situ on upstaging and outcome following radical cystectomy for bladder cancer. World J. Urol..

[21] Bouchelouche K. (2022). PET/CT in Bladder Cancer: An Update. Semin. Nucl. Med..

[22] Mertens L.S., Fioole-Bruining A., Vegt E., Vogel W.V., van Rhijn B.W., Horenblas S. (2013). Impact of (18) F-fluorodeoxyglucose (FDG)-positron-emission tomography/computed tomography (PET/CT) on management of patients with carcinoma invading bladder muscle. BJU Int..

[23] Tufano A., Rosati D., Moriconi M., Santarelli V., Canale V., Salciccia S., Sciarra A., Franco G., Cantisani V., Di Pierro G.B. (2024). Diagnostic Accuracy of Contrast-Enhanced Ultra-sound (CEUS) in the Detection of Muscle-Invasive Bladder Cancer: A Systematic Review and Diagnostic Meta-Analysis. Curr. Oncol..

[24] Amenyogbe A., Lemire F., Yachnin D., Carrier M., McAlpine K., Breau R.H., Bossé D., Wang T.-F., Morash C., Cagiannos I. (2022). A survey of physician perception and practices regarding pharmacological thromboprophylaxis during chemotherapy for bladder cancer. Can. Urol. Assoc. J..

[25] Proietti F., Flammia R.S., Licari L.C., Bologna E., Bove A.M., Brassetti A., Tuderti G., Mastroianni R., Tufano A., Simone G. (2024). Impacts of Neoadjuvant Chemotherapy on Perioperative Outcomes in Patients with Bladder Cancer Treated with Radical Cystectomy: A Single High-Volume Center Experience. J. Pers. Med..

[26] de Angelis M., Jannello L.M.I., Siech C., Baudo A., Di Bello F., Goyal J.A., Tian Z., Longo N., de Cobelli O., Chun F.K. (2025). Neoadjuvant chemotherapy before radical cystectomy in patients with organ-confined and non-organ-confined urothelial carcinoma. Urol. Oncol..

[27] Audenet F., Sfakianos J.P., Waingankar N., Ruel N.H., Galsky M.D., Yuh B.E., Gin G.E. (2018). A delay ≥8 weeks to neoadjuvant chemotherapy before radical cystectomy increases the risk of upstaging. Urol. Oncol. Semin. Orig. Investig..

[28] Liu W.J., Campbell R.A., Michael P.D., Wood A., Haywood S.C., Eltemamy M., Kaouk J., Campbell S.C., Haber G.-P., Weight C.J. (2024). Clinical Upstaging After Neoadjuvant Chemotherapy Impacting Eligibility for Vaginal-sparing Cystectomy: Identifying Bladder Cancer Patients Who May Benefit From Interim Imaging. Urology.

[29] Boeri L., Soligo M., Frank I., Boorjian S.A., Thompson R.H., Tollefson M., Tarrel R., Quevedo F.J., Cheville J.C., Karnes R.J. (2019). Clinical predictors and survival outcome of patients receiving suboptimal neoadjuvant chemotherapy and radical cystectomy for muscle-invasive bladder cancer: A single-center experience. World J. Urol..

[30] Balar A.V., Castellano D., O’Donnell P.H., Grivas P., Vuky J., Powles T., Plimack E.R., Hahn N.M., de Wit R., Pang L. (2017). First-line pembrolizumab in cisplatin-ineligible patients with locally advanced and unresectable or metastatic urothelial cancer (KEYNOTE-052): A multicentre, single-arm, phase 2 study. Lancet Oncol..

[31] Liu K.X., Haas-Kogan D., Laprie A. (2022). Role of Radiotherapy in the Era of Targeted Therapy and Precision Oncology.

[32] Fan X., He W., Huang J. (2023). Bladder-sparing approaches for muscle invasive bladder cancer: A narrative review of current evidence and future perspectives. Transl. Androl. Urol..

[33] Renner A., Burotto M., Valdes J.M., Roman J.C., Walton-Diaz A. (2021). Neoadjuvant immunotherapy for muscle invasive urothelial bladder carcinoma: Will it change current standards?. Ther. Adv. Urol..

[34] Robertson A.G., Meghani K., Cooley L.F., McLaughlin K.A., Fall L.A., Yu Y., Castro M.A.A., Groeneveld C.S., de Reyniès A., Nazarov V.I. (2023). Expression-based subtypes define pathologic response to neoadjuvant immune-checkpoint inhibitors in muscle-invasive bladder cancer. Nat. Commun..

[35] Moschini M., Gandaglia G., Dehò F., Salonia A., Briganti A., Montorsi F. (2022). Bladder cancer: ESMO Clinical Practice Guideline for diagnosis, treatment and follow-up. Ann. Oncol..

[36] Zhu Z., Xiao Y., Hu S., Wang Z., Zhu Z. (2022). Neoadjuvant and Adjuvant Chemotherapy for Variant Histology Bladder Cancers: A Systematic Review and Meta-Analysis. Front. Oncol..

[37] Campbell R.A., Khanna A., Boorjian S.A., Knorr J., Cox R., Nicholas M., Cheville J., Sharma V., Murthy P.B., Tarrell R. (2023). Impact of Neoadjuvant Chemotherapy on Pathologic Downstaging in Patients with Variant Histology Undergoing Radical Cystectomy. Clin. Genitourin. Cancer.

[38] Tabayoyong W., Li R., Gao J., Kamat A. (2018). Optimal Timing of Chemotherapy and Surgery in Patients with Muscle-Invasive Bladder Cancer and Upper Urinary Tract Urothelial Carcinoma. Urol. Clin. N. Am..

[39] Krasnow R.E., Drumm M., Roberts H.J., Niemierko A., Wu C.-L., Wu S., Zhang J., Heney N.M., Wszolek M.F., Blute M.L. (2017). Clinical Outcomes of Patients with Histologic Variants of Urothelial Cancer Treated with Trimodality Bladder-sparing Therapy. Eur. Urol..

[40] Fischer-Valuck B.W., Michalski J.M., Contreras J.A., Brenneman R., Christodouleas J.P., Abraham C.D., Kim E.H., Arora V.K., Bullock A.D., Carmona R. (2018). A propensity analysis comparing definitive chemo-radiotherapy for muscle-invasive squamous cell carcinoma of the bladder vs. urothelial carcinoma of the bladder using the National Cancer Database. Clin. Transl. Radiat. Oncol..

